# Evaluating the Quality and Readability of Generative Artificial Intelligence (AI) Chatbot Responses in the Management of Achilles Tendon Rupture

**DOI:** 10.7759/cureus.78313

**Published:** 2025-01-31

**Authors:** Christopher E Collins, Peter A Giammanco, Monica Guirgus, Mikayla Kricfalusi, Richard C Rice, Rusheel Nayak, David Ruckle, Ryan Filler, Joseph G Elsissy

**Affiliations:** 1 Orthopedic Surgery, California University of Science and Medicine, Colton, USA; 2 Orthopedic Surgery, Arrowhead Regional Medical Center, Colton, USA; 3 Department of Orthopedic Surgery, Loma Linda University Medical Center, Loma Linda, USA; 4 Foot and Ankle, Hospital for Special Surgery, New York City, USA; 5 Foot and Ankle, Northwestern University, Chicago, USA

**Keywords:** achilles tear, artificial intelligence, deep learning, orthopedics, sports medicine

## Abstract

Introduction: The rise of artificial intelligence (AI), including generative chatbots like ChatGPT (OpenAI, San Francisco, CA, USA), has revolutionized many fields, including healthcare. Patients have gained the ability to prompt chatbots to generate purportedly accurate and individualized healthcare content. This study analyzed the readability and quality of answers to Achilles tendon rupture questions from six generative AI chatbots to evaluate and distinguish their potential as patient education resources.

Methods: The six AI models used were ChatGPT 3.5, ChatGPT 4, Gemini 1.0 (previously Bard; Google, Mountain View, CA, USA), Gemini 1.5 Pro, Claude (Anthropic, San Francisco, CA, USA) and Grok (xAI, Palo Alto, CA, USA) without prior prompting. Each was asked 10 common patient questions about Achilles tendon rupture, determined by five orthopaedic surgeons. The readability of generative responses was measured using Flesch-Kincaid Reading Grade Level, Gunning Fog, and SMOG (Simple Measure of Gobbledygook). The response quality was subsequently graded using the DISCERN criteria by five blinded orthopaedic surgeons.

Results: Gemini 1.0 generated statistically significant differences in ease of readability (closest to average American reading level) than responses from ChatGPT 3.5, ChatGPT 4, and Claude. Additionally, mean DISCERN scores demonstrated significantly higher quality of responses from Gemini 1.0 (63.0±5.1) and ChatGPT 4 (63.8±6.2) than ChatGPT 3.5 (53.8±3.8), Claude (55.0±3.8), and Grok (54.2±4.8). However, the overall quality (question 16, DISCERN) of each model was averaged and graded at an above-average level (range, 3.4-4.4).

Discussion and conclusion: Our results indicate that generative chatbots can potentially serve as patient education resources alongside physicians. Although some models lacked sufficient content, each performed above average in overall quality. With the lowest readability and highest DISCERN scores, Gemini 1.0 outperformed ChatGPT, Claude, and Grok and potentially emerged as the simplest and most reliable generative chatbot regarding management of Achilles tendon rupture.

## Introduction

Modern society has progressively incorporated computer networks in an attempt to automate and enhance nearly every facet of life, including patient education. However, internet healthcare information sources are highly variable and may even be misleading, therefore negatively impacting patient decision-making [1]. Additionally, the average readability across many websites has been demonstrated to be at a ninth-grade level, above that of the average American [2]. However, health literacy is recognized as key to informed consent regarding procedure choice and thus objective, unbiased, and literary-level appropriate patient education resources are essential to enhance generalizability and accurately equip patients to make informed medical decisions [2,3]. 

Artificial intelligence (AI) has revolutionized the delivery of healthcare and education. Generative AI, such as ChatGPT (OpenAI, San Francisco, CA, USA), are interactive, easy to use, and readily available to the public [4,5]. These AI chatbots represent an accessible and powerful, yet unvalidated alternative online patient education resource. Previous research has highlighted AI’s generalizability, accuracy, and individualized production of education materials. AI has been reported to explain surgical procedures and outcomes in terms that allow for better patient understanding following prompting [6]. Additionally, Kirchner et al. in 2023 found that ChatGPT was able to effectively rewrite orthopaedic patient education materials regarding common injuries and surgeries, while maintaining factual information and acceptable detail [7].

Achilles tendon rupture (ATR) is a common orthopedic injury, representing nearly 20% of large tendon injuries and being the most frequently ruptured tendon in the lower limb [8,9]. Despite its prevalence, management of ATR remains controversial, with treatment options ranging from conservative to surgical based on factors such as activity level, age, goals of care, tear chronicity, extent of tear (partial versus complete), and patient preference [10,11]. Given the variability in treatment approaches, it is crucial for patients to fully understand the different options and their outcomes to make informed decisions. Patients often seek additional information from sources beyond their physician, such as internet articles, though the quality of these sources can be concerning. This study aims to analyze the quality and readability of responses to common ATR-related questions provided by AI chatbots, including ChatGPT 3.5, ChatGPT 4, Gemini 1.0 (Google, Mountain View, CA, USA), Gemini 1.5 Pro, (Anthropic, San Francisco, CA, USA) and Grok (xAI, Palo Alto, CA, USA). We evaluated differences in quality and readability among these chatbots for ATR patient education.

This article was previously presented as a podium presentation at the Clinical Orthopaedic Society 112th Annual Meeting on September 6, 2024, in Tampa, Florida and as a poster at the Orthopaedic Summit Evolving Techniques 2024 Meeting on September 13-14, 2024 in Las Vegas, Nevada. 

## Materials and methods

ChatGPT 3.5 (last update September 2021), ChatGPT 4 (last update April 2023), Gemini 1.0 (continuously updated) and Gemini 1.5 Pro (continuously updated), Claude (last update April 2024), and Grok (last update November 2023) were all accessed in April 2024 and tasked with answering 10 common patient questions regarding ATR and prospective treatment options. These AI models were chosen based on the following criteria. To adequately account for access, both paid and free chatbots were evaluated: three AI bots require payment (ChatGPT 4, Gemini 1.5 Pro, and Grok), while the other three (ChatGPT 3.5, Gemini 1.0, and Claude) are free to use. Both newer and older generation models as the date of last knowledge update among the models varies. Newer generation models (ChatGPT 4, Gemini 1.5 Pro, Grok) have access to more recent data upon which to provide answers to users' questions, potentially providing more accurate and reliable answers. 

All questions were written at the average American reading level of eighth grade as verified by Flesh-Kincaid Reading Grade Level [12]. The questions were curated in consultation with five orthopaedic surgeons based on their experience with common questions and concerns among patients with ATR. These were the same five orthopaedic surgeons that would eventually grade the answers to these questions from each AI model using the DISCERN criteria. The 10 questions were posed in a separate new chat with each of the AI models and their answers were recorded in a Word document (Microsoft, Redmond, WA, USA). None of the AI models were trained in any fashion beforehand.

Readable (Added Bytes Ltd., Brighton, UK), an online toolkit containing multiple assessments of a text's readability, was used to provide the readability score of an AI model’s answers [13,14]. Readability was assessed across three commonly used metrics: Flesch-Kincaid Reading Grade Level, Gunning Fog Score, and SMOG (Simple Measure of Gobbledygook) score [15,16]. For each of these, lower scores correlate to a lower reading grade level. Due to the differing scales of each readability formula, scores were transformed to a Z-score within SPSS (IBM Corp., Armonk, NY, USA) in order to standardize the scales and allow for comparison [17]. 

The answers of each AI model were then placed in a blinded Google Form, followed by the 16 DISCERN criteria questions. The identifying information of each AI model was blinded from the five orthopaedic surgeons that scored the quality of the answers using the DISCERN criteria. The DISCERN criteria is made up of 16 questions and is used to assess consumer health information’s quality and reliability [18]. The first eight questions address the reliability of the publication and whether or not it can be trusted as a source of information to inform treatment choices. The next seven questions focus on the details of treatment options discussed. The final question is an overall quality rating of the source as a whole. Each question is graded on a scale from 1 to 5, thus the maximum possible score for an AI model was 80. A score of “1” meant that the AI model did not fulfill the DISCERN criteria question. A score of “5” meant that the AI model completely fulfilled the DISCERN criteria question. Scores of “2-4” meant that the AI model partially fulfilled the DISCERN criteria question and was left to the grader’s judgment on how low or high they felt the criteria was fulfilled, with a score of “3” being average. There is not a consensus on the interpretation of total DISCERN scores in the literature [19]. However, Hurley et al. 2024 provided the following subdivisions: a score of 70-80 correlates to “excellent” information quality and reliability, while a score of 50-69 is deemed “good” [20].

The mean reading grade level (RGL) was combined between the three readability formulas and then compared for each AI model using a Kruskal-Wallis test. The Kruskal-Wallis test was also used to determine statistical significance between DISCERN scores of each AI model with a difference meaning that the source of information is of higher quality. All statistical analyses were performed using SPSS statistics for Windows, version 28.0.1.0 (142). This study received Institutional Review Board exempt status as no patients were involved.

## Results

Ten questions were each asked to ChatGPT 3.5, ChatGPT 4, Gemini 1.0, Gemini 1.5 Pro, Claude, and Grok. An example of an answer from each AI model is shown below (Table 1).

**Table 1:** Example answers from each AI model to a common question

Question: Who should consider Achilles tendon repair surgery?	
ChatGPT 3.5	“Achilles tendon repair surgery is typically considered for individuals who have experienced a complete tear (rupture) of the Achilles tendon, particularly if they are physically active or rely on their feet for work or daily activities. Candidates for Achilles tendon repair surgery often include: 1. Athletes: Especially those involved in sports that require quick bursts of activity or jumping, such as basketball, soccer, or tennis. 2. Active individuals: People who engage in regular physical activity and want to restore full function to their ankle. 3. Individuals with severe injuries: Those who have sustained a significant tear or rupture of the Achilles tendon that affects their ability to walk or perform daily tasks. 4. Failed conservative treatment: Patients who have tried non-surgical treatments such as physical therapy and immobilization without success. 5. Those with high physical demands: People whose occupations or lifestyles require a high level of physical activity, such as manual laborers or military personnel. Ultimately, the decision to undergo Achilles tendon repair surgery should be made in consultation with a healthcare professional, considering factors such as the severity of the injury, the individual's overall health, and their goals for recovery and return to activity.”
ChatGPT 4	“Achilles tendon repair surgery is considered for individuals who have damaged or ruptured their Achilles tendon, which is a strong fibrous cord that connects the muscles in the back of your calf to your heel bone. This type of surgery is usually recommended for: Athletes and Active Individuals: Especially those who require full strength and functionality of their Achilles tendon for running, jumping, or other activities. Complete Ruptures: If the tendon has completely torn or ruptured, surgery is often necessary to reattach the ends of the tendon. Partial Tears Not Responding to Conservative Treatment: In cases where non-surgical treatments (such as physical therapy, rest, and immobilization) have not led to improvement or complete healing. Individuals Seeking Faster Recovery: Surgery may be considered to potentially shorten the recovery time compared to conservative treatment, depending on the individual's overall health and specific circumstances. Patients with Recurrent Tendon Injuries: Those who have a history of Achilles tendon issues may benefit from surgery to address underlying problems and prevent future injuries. Young and Healthy Individuals: These patients tend to have better outcomes with surgery given their generally faster healing and recovery capabilities. The decision to undergo Achilles tendon repair surgery should be made after a thorough evaluation and discussion with an orthopedic surgeon or a specialist in sports medicine. They will consider factors such as the severity of the injury, the individual's lifestyle and activity level, age, general health, and the potential benefits and risks of surgery. Non-surgical options are also viable for many cases, especially for less severe injuries, older patients, or those with significant health issues that may complicate surgery and recovery.”
Gemini 1.0	“Achilles tendon repair surgery is typically recommended for individuals with a complete rupture of their Achilles tendon. This can cause a significant loss of function and strength in the ankle and calf, making it difficult to walk, run, or jump. Here are some of the factors a doctor will consider when deciding if Achilles tendon repair surgery is right for you: Severity of the tear: A complete rupture of the Achilles tendon is more likely to require surgery than a partial tear. Age and activity level: Younger, more active individuals are generally better candidates for surgery than older adults or those who are less active. Overall health: Certain medical conditions, such as diabetes or peripheral vascular disease, can increase the risk of complications from surgery. Previous treatment: If non-surgical treatment, such as immobilization in a cast or boot, has not been successful in healing the tendon, then surgery may be an option. If you are considering Achilles tendon repair surgery, it is important to discuss the risks and benefits of the procedure with your doctor.”
Gemini 1.5 Pro	“Achilles Tendon Repair Surgery: Who Needs It? Achilles tendon repair surgery is typically recommended for individuals experiencing a complete or near-complete tear of the Achilles tendon. This injury often occurs during sports or activities that involve sudden stops and starts, jumping, or forceful pushing off of the foot. Here's a breakdown of who might need this surgery: Individuals with a complete or near-complete Achilles tendon tear: Symptoms: Sudden sharp pain in the back of the ankle, often described as a "pop," followed by difficulty walking, rising on the toes, and pushing off the injured leg. Diagnosis: Physical examination and imaging tests like an MRI or ultrasound can confirm the tear and its severity. Other factors influencing the decision for surgery: Age and activity level: Younger, active individuals are often good candidates for surgery to regain full function and mobility. Overall health: Individuals with good overall health are better suited to tolerate surgery and recover successfully. Severity of the tear and time since the injury: Acute tears tend to have better surgical outcomes compared to chronic tears. Alternatives to surgery: Non-surgical treatment: This may be suitable for minor tears or for individuals who are not good candidates for surgery. It usually involves immobilization, physical therapy, and pain management. Important considerations: Surgical risks: Like any surgery, Achilles tendon repair has potential risks such as infection, bleeding, blood clots, nerve damage, and re-rupture of the tendon. Recovery time: Recovery is a lengthy process, typically involving several months of rehabilitation to regain strength and flexibility. It's crucial to consult with an orthopedic surgeon or foot and ankle specialist to determine the best course of treatment based on individual circumstances. They can evaluate the extent of the injury, discuss potential risks and benefits of surgery, and recommend the most appropriate treatment plan.”
Claude	“Achilles tendon repair surgery, also known as Achilles tendon reconstruction or repair, is typically recommended for individuals who have suffered from a complete or partial tear of the Achilles tendon. The Achilles tendon is the large tendon that connects the calf muscles to the heel bone and plays a crucial role in walking, running, and jumping. Here are some common situations where Achilles tendon repair surgery may be considered: 1. Acute Achilles tendon rupture: This is a complete tear or rupture of the Achilles tendon, often resulting from a sudden forceful movement, such as during sports activities or a fall. Surgery is usually recommended to reattach the torn ends of the tendon and restore the function of the calf muscles. 2. Chronic Achilles tendon tear or degeneration: In some cases, the Achilles tendon can degenerate over time due to repetitive stress or overuse, leading to partial or complete tears. Surgery may be recommended to repair the damaged tendon and alleviate pain and disability. 3. Delayed or neglected Achilles tendon rupture: If an Achilles tendon rupture is not treated promptly, the torn ends of the tendon may separate and retract, making it more difficult to repair. In such cases, surgery may be necessary to reattach the tendon and restore function. 4. Failed conservative treatment: In some cases, non-surgical treatments, such as immobilization, physical therapy, or other conservative measures, may not be effective in healing an Achilles tendon injury. Surgery may be considered if the tendon does not heal properly or if the individual continues to experience significant pain or functional limitations. It's important to note that the decision to undergo Achilles tendon repair surgery is typically made in consultation with an orthopedic surgeon or a sports medicine specialist. They will evaluate the extent of the injury, the individual's age, activity level, and overall health to determine the most appropriate treatment plan.”
Grok	“Individuals who should consider Achilles tendon repair surgery are those who have experienced a severe Achilles tendon injury, such as a full rupture, or those with chronic Achilles tendon injuries that haven't responded well to conservative treatments. Surgery is often recommended for tendon tears that are larger than half the width of the tendon. The decision for surgery may also depend on factors such as the patient's age, activity level, and the severity and nature of the injury. Additionally, individuals who are experiencing persistent pain, weakness, or functional limitations due to Achilles tendon issues may be candidates for surgery.”

Reading grade level (RGL)

**Figure 1:**
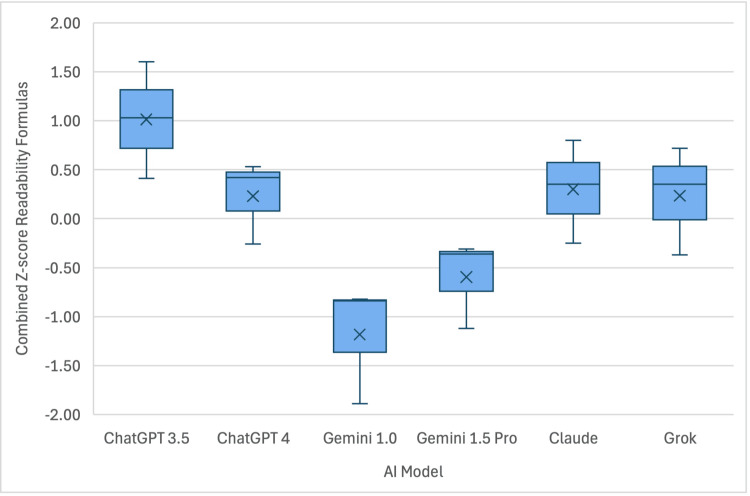
Box and whisker plot demonstrating the readability score for each AI model AI - Artificial Intelligence

The mean RGL of the 10 questions provided to the AI models by five orthopaedic surgeons was approximately an eighth-grade reading level (Table 2), readable by roughly 85% of the general public in the United States [12]. Responses from ChatGPT 3.5 had mean RGLs of 15.23, 18.19, and 16.70 on Flesch-Kincaid, Gunning Fog, and SMOG, respectively. ChatGPT 4 performed slightly better with means of 13.38, 15.38, and 15.03, respectively. Gemini 1.0 yielded mean RGL scores of 9.60, 12.09, and 12.07, respectively. Gemini 1.5 Pro produced mean values of 11.40, 13.15, 13.31, respectively. Claude demonstrated mean values of 13.65, 16.08, and 15.02, respectively. Grok had mean RGLs of 13.23, 15.94, and 14.98, respectively (Table 3). A combined analysis of the three readability formulas using a Z-score is represented in Figure 1.

**Table 2:** Readability formula descriptive statistics for 10 questions answered by AI models SD - Standard Deviation Q1 - 1st Quartile Q3 - 3rd Quartile SMOG - Simple Measure of Gobbledygook

Readability Formula	Mean +/- SD	Median (Q1-Q3)	Minimum	Maximum	Range
Flesch-Kincaid	8.45±1.55	8.30 (7.25-9.93)	6.30	10.70	4.40
Gunning Fog	8.68±2.74	8.15 (8.00-8.50)	3.60	14.20	10.60
SMOG	8.71±2.21	8.80 (8.80-8.80)	3.10	11.20	8.10

**Table 3:** Descriptive statistics for readability formulas of answers by AI model SD - Standard Deviation Q1 - 1st Quartile Q3 - 3rd Quartile SMOG - Simple Measure of Gobbledygook

AI Model	Reading Formula	Mean +/- SD	Median (Q1-Q3)	Minimum	Maximum	Range
ChatGPT 3.5	Flesch-Kincaid	15.23±1.73	15.65 (14.05-16.40)	12.70	17.90	5.20
	Gunning Fog	18.19±2.42	18.40 (17.60-19.38)	13.50	22.00	8.50
	SMOG	16.70±1.46	16.80 (16.33-17.58)	14.20	19.00	4.80
ChatGPT 4	Flesch-Kincaid	13.38±1.81	13.00 (12.38-14.10)	10.40	16.70	6.30
	Gunning Fog	15.38±2.23	15.35 (13.53-17.20)	12.40	18.90	6.50
	SMOG	15.03±1.33	14.50 (14.25-15.75)	13.10	17.60	4.50
Gemini 1.0	Flesch-Kincaid	9.60±1.94	8.65 (8.33-10.55)	7.40	13.20	5.80
	Gunning Fog	12.09±2.04	11.30 (10.85-12.38)	10.00	15.80	5.80
	SMOG	12.07±1.51	11.45 (11.05-12.60)	10.60	14.90	4.30
Gemini 1.5 Pro	Flesch-Kincaid	11.40±1.49	11.75 (10.85-12.25)	8.20	13.40	5.20
	Gunning Fog	13.15±1.77	13.25 (12.20-13.95)	10.30	16.50	6.20
	SMOG	13.31±1.13	13.40 (12.78-13.88)	11.00	15.10	4.10
Claude	Flesch-Kincaid	13.65±1.83	13.75 (12.85-14.68)	10.00	16.70	6.70
	Gunning Fog	16.08±1.63	15.80 (15.23-16.98)	13.50	18.70	5.20
	SMOG	15.02±1.22	15.00 (14.45-15.73)	12.70	16.90	4.20
Grok	Flesch-Kincaid	13.23±2.54	12.30 (11.13-15.13)	10.90	17.90	7.00
	Gunning Fog	15.94±2.90	15.15 (13.63-17.55)	13.10	21.80	8.70
	SMOG	14.98±2.04	14.10 (13.73-16.40)	12.60	19.10	6.50

Kruskal-Wallis testing revealed a significant difference between groups (p=0.032). Post hoc analysis revealed a significant difference between Gemini 1.0 and ChatGPT 3.5 (p=0.003), Gemini 1.0 and ChatGPT 4 (p=0.039), Gemini 1.0 and Claude (p=0.032), and Gemini 1.5 Pro and ChatGPT 3.5 (p=0.014). Of note, for each AI model, the answers to the 10 questions averaged above the eighth-grade reading level (Table 3).

DISCERN score

**Figure 2:**
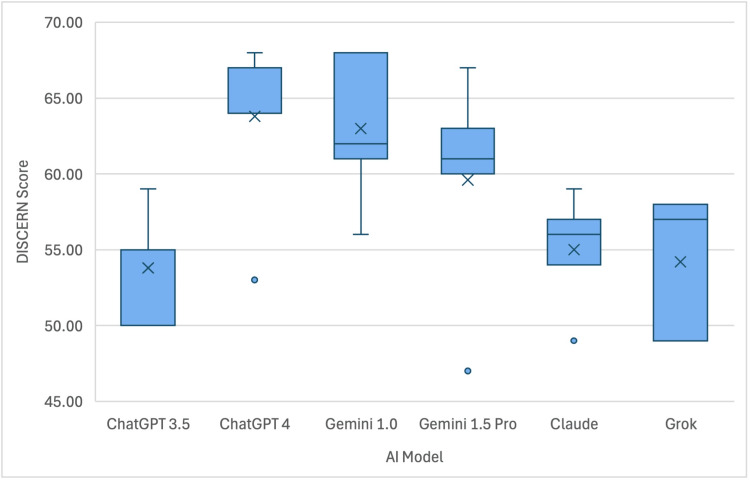
Box and whisker plot demonstrating the readability score for each AI model AI - Artificial Intelligence

Upon statistical analysis utilizing an independent sample Kruskal-Wallis test, a significant difference in mean DISCERN scores between groups was found (p=0.031, Table 4). Post hoc analysis demonstrated that these statistically significant differences lied among the following pairs: Gemini 1.0 (mean: 63.00, range: 56.00-68.00) and ChatGPT 3.5 (mean: 53.80, range: 50.00-59.00) (p=0.02), Gemini 1.0 and Grok (mean: 54.20, range: 49.00-58.00) (p=0.027), Gemini 1.0 and Claude (mean: 55.00, range: 49.00-59.00) (p=0.035), ChatGPT 4 (mean: 63.80, range: 53.00-68.00) and ChatGPT 3.5 (p=0.02), ChatGPT 4 and Grok (p=0.027), and ChatGPT 4 and Claude (p=0.035). Visual representation of the scores can be seen in Figure 2.

**Table 4:** Descriptive statistics of DISCERN criteria score by AI model SD - Standard Deviation Q1 - 1st Quartile Q3 - 3rd Quartile AI - Artificial Intelligence

Al Model	Mean +/- SD	Median (Q1-Q3)	Minimum	Maximum	Range
Chat GPT 3.5	53.80±3.83	55.00 (50.00-55.00)	50.00	59.00	9.00
Chat GPT 4	63.80±6.22	67.00 (64.00-67.00)	53.00	68.00	15.00
Gemini 1.0	63.00±5.10	62.00 (61.00-68.00)	56.00	68.00	12.00
Gemini 1.5 Pro	59.60±7.54	61.00 (60.00-63.00)	47.00	67.00	20.00
Claude	55.00±3.81	56.00 (54.00-57.00)	49.00	59.00	10.00
Grok	54.20±4.76	57.00 (49.00-58.00)	49.00	58.00	9.00

With respect to individual DISCERN criteria questions, questions 4 and 5, which inquire about source citation, were consistently graded lowest with respect to the other 14 questions. The scores for questions 4 and 5, respectively, were as follows: ChatGPT 3.5 (1.2, 1.6), ChatGPT 4.0 (2.6, 2.4), Gemini 1.0 (3.2, 2.8), Gemini 1.5 Pro (1.8, 1.8), Claude (1, 1.2), and Grok (1.6, 1.4). However, the overall quality of each AI model (question 16, Table 6) was graded as above average. The average scores were as follows: ChatGPT 3.5 (3.4), ChatGPT 4.0 (4.2), Gemini 1.0 (3.8), Gemini 1.5 Pro (4.4), Claude (3.8), and Grok (3.6).

## Discussion

This study assessed the readability and quality of patient health information provided by six AI models in response to common ATR questions (Appendix 1). Readability was evaluated using three formulas: Flesch-Kincaid Reading Grade Level, Gunning Fog, and SMOG. Analysis revealed that Gemini 1.0 had a significantly lower reading level than ChatGPT 3.5, ChatGPT 4, and Claude, with no significant differences among the latter three models (Figure 1). Gemini 1.5 Pro was easier to read than ChatGPT 3.5, but no differences were observed between Grok and the other models. Quality and reliability were assessed using the DISCERN criteria (Appendix 2), showing that Gemini 1.0 and ChatGPT 4 outperformed ChatGPT 3.5, Grok, and Claude (Figure 2). Thus, Gemini 1.0 and ChatGPT 4 may be the best options for high-quality patient education on ATR.

Previous studies have examined the quality of information provided by AI models, without consensus on their use in patient education [13,21-25]. This study highlights the readability of generative AI. Although Gemini 1.0 was the most readable, all six AI models produced responses rated above the eighth-grade level (standard for average Americans) on all readability formulas [2]. Prior studies confirm that high readability levels can limit the utility of such material [13,21-29]. However, users can prompt AI to simplify responses, such as by asking for an explanation at an eighth-grade level. Studies show that AI can adjust output based on such prompts, though results regarding response quality vary [7,23,30]. This study used default-level responses for consistency, acknowledging that adjustable options are available.

This study found that Gemini 1.0 and ChatGPT 4 provide high-quality information regarding ATR. However, all of the models tested are still lacking in critical areas. The DISCERN criteria questions regarding information source and publication date (questions 4 and 5) scored consistently lower across each AI model. Of the 12 possible opportunities, only one time did an AI model score higher than 3 on average (3.2, Gemini 1.0, question 4). The rest of the scores for questions 4 and 5 were approximately 1 or 2, reflecting the consistent lack of source citation. Similar findings have been reported on ChatGPT’s responses to hand surgery questions, including lack of source citation and low reliability per the DISCERN criteria [13,21-25]. Future AI models should incorporate reference lists to enhance credibility.

The results of the present study may ease concerns about AI providing misinformation, as the average score of question 16 (overall quality as a source of information) was 3.4 or higher across each AI model, as rated by the five orthopaedic respondents. This highlights that although the majority of models scored poorly on the respective DISCERN questions regarding sourcing, the generated information of each AI model was still validated as above average quality sources of medical information by professionals in the field. 

The study highlights both strengths and weaknesses in the evaluated AI models and proposes potential solutions. For example, although readability analysis revealed that AI models often provide information at a high reading level, users can request answers at a specific grade level [2]. Additionally, while source citations were lacking, orthopaedist respondents confirmed that the quality of the medical information was still above average [26,27]. Nonetheless, generative AI models could be improved in terms of readability and the quality of medical information on ATR [28,29].

Limitations of this study include a small number of orthopaedic surgeons grading the AI models, which increases the risk for bias in the results. Additionally, each of the graders had varying experience, ranging from four to 20 years of practice. Another limitation is that the questions posed to AI models were representative of patient questions as provided by orthopaedic surgeons based on their own experiences, rather than what patients may want to ask an AI model on their own. Finally, the orthopaedic graders who created the 10 questions also graded the quality of responses, further increasing the risk of bias. 

Thus, after considering readability, quality, and access, we believe that Gemini 1.0 serves as the strongest and most appropriate AI model for patients to consult about ATR, given the current state of these six AI models at the time of publication.

## Conclusions

This study determined the quality, reliability, and readability of patient education provided by six different AI models regarding ATR. As AI continues to rapidly evolve and play an ever-increasing role in daily life, the importance of ensuring reliably sourced and accurate information to patients will become more crucial. All models have room for improvement regarding citation of reliable sources in generation of their answers. However, we identify potentially the strongest model for providing patient health information regarding ATR-Gemini 1.0. Overall, generative AI language models can play a pivotal role in increasing a patient's understanding of their Achilles injury and treatment options when used in conjunction with their orthopaedic surgeon.

## Appendices

Appendix 1

**Table 5:** Common Achilles tendon rupture questions

No.	Question
1.	Should I get Achilles tendon repair surgery?
2.	Who should consider Achilles tendon repair surgery?
3.	Can I avoid Achilles tendon repair surgery?
4.	How do doctors perform Achilles tendon repair surgery?
5.	What will the recovery be like after Achilles tendon repair surgery?
6.	Can I play sports again after Achilles tendon repair surgery?
7.	When can I go back to work after Achilles tendon repair surgery?
8.	What problems might happen after Achilles tendon repair surgery?
9.	What is the success rate of Achilles tendon repair surgery?
10.	What are the treatment options for Achilles tendon rupture?

Appendix 2

**Table 6:** DISCERN criteria questions

No.	Question
1.	Are the aims clear?
2.	Does it achieve its aims?
3.	Is it relevant?
4.	Is it clear what sources of information were used to compile the publication (other than the author or producer)?
5.	Is it clear when the information used or reported in the publication was produced?
6.	Is it balanced and unbiased?
7.	Does it provide details of additional sources of support and information?
8.	Does it refer to areas of uncertainty?
9.	Does it describe how each treatment works?
10.	Does it describe the benefits of each treatment?
11.	Does it describe the risks of each treatment?
12.	Does it describe what would happen if no treatment is used?
13.	Does it describe how the treatment choices affect overall quality of life?
14.	Is it clear that there may be more than one possible treatment choice?
15.	Does it provide support for shared decision-making?
16.	Based on the answers to all of the above questions, rate the overall quality of the publication as a source of information about treatment choices.
